# Acute Visual Loss Induced by Dexamethasone During Neoadjuvant Docetaxol

**DOI:** 10.4137/cmo.s339

**Published:** 2008-02-09

**Authors:** D.L. Gregory, C.D. Jones, E.R.E. Denton, A.N. Harnett

**Affiliations:** 1 Department of Oncology, Addenbrookes Hospital, Cambridge, UK; 2 Department of Ophthalmology, Norfolk and Norwich University Hospital Norwich, UK; 3 Department of Radiology, Norfolk and Norwich University Hospital Norwich, UK; 4 Department of Oncology, Norfolk and Norwich University Hospital Norwich, UK

**Keywords:** central serous retinopathy, taxanes, docetaxol, steroid premedication, dexamethasone

## Abstract

We present a case of a female patient who developed acute onset of visual loss due to central serous retinopathy as a consequence of steroid premedication for docetaxol given as second line neoadjuvant chemotherapy for breast cancer. Central serous retinopathy is a recognised association with steroids but has not been previously reported in association with the management of solid tumours. Reduction in steroid dose and duration permitted recovery of her visual acuity while allowing completion of the prescribed chemotherapy regimen. An overview of the presentation, pathogenesis, aetiologies and management of central serous retinopathy is given.

## Case History

### History

A 42 year old female presented with several weeks history of a lump in the left breast. She had a history of congenital dislocation of the hip, menorrhagia investigated with normal laparoscopy and had been treated for a dry eye syndrome of unknown aetiology. She had previously been treated for anxiety and depression. She was not on any medications. She was premenopausal and had 2 children both delivered by caesarean section. She had no significant family history.

### Examination

Examination revealed a 6 by 7 cm mass in the upper outer quadrant of the left breast extending to the areola. There was associated skin tethering but no fixation to deeper structures. There were mobile lymph nodes palpable in the medial aspect of the left axilla. The remainder of the examination was unremarkable. Her clinical staging was T4bN1M0.

### Investigations

Mammography and ultrasound confirmed a malignant appearing lesion at least 6 cm in diameter and ultrasound guided core biopsy showed invasive lobular carcinoma which was ER positive. Baseline liver and renal function tests, calcium and full blood count were normal other than a mild iron deficiency anaemia, which was thought secondary to menorrhagia. Chest radiograph and liver ultrasound showed no evidence of metastatic disease. MRI confirmed a 7 cm area of malignant appearing enhancement in the left breast, with no abnormality in axilla or contra lateral breast. (Fig. 1a)

### Management

In view of the locally advanced nature of her carcinoma she was offered neoadjuvant chemotherapy with FEC (5 fluorouracil 600 mg/m^2^, epirubicin 60 mg/m^2^ and cyclophosphamide 600 mg/m^2^).

### Progress

After 3 cycles of chemotherapy there had been little improvement in the size of the lump on clinical examination. A further MRI (Fig. 1b) showed no change in the malignant enhancement within the left breast. A new area of malignant type enhancement was noted within the right breast which was impalpable. She proceeded with 2nd line neoadjuvant chemotherapy using docetaxol (100 mg/m^2^) which was given with dexamethasone cover for 72 hours commencing 24 hours before each cycle at a dose of 8 mg twice daily.

Following the 2nd cycle she was re examined and the lesion in the left breast had shrunk to 4 cm by 5 cm and was now poorly defined. There was no palpable lesion in the right breast. After the 3rd cycle she developed acute loss of visual acuity in the left eye such that she was only able to count fingers, vision in the right eye was unaffected. The differential diagnosis included choroidal or retinal metastasis, retinal detachment, or other retinal pathology. Indirect opthalmoscopy revealed sub macular fluid and fluorescein angiography (Fig. 2a), (Fig. 2b) and (Fig. 2c) showed the very rare but classical “smoking stack” appearance. A diagnosis of steroid induced central serous retinopathy was therefore made. After discussion with the patient and ophthalmologist the decision was made to reduce the duration of steroid premedication to 24 hours only. On this regime she completed the course of 6 cycles of docetaxol without deterioration in her visual symptoms and without further incident. A further MRI was performed (Fig. 1c) which showed an excellent response to treatment and this was confirmed clinically. She proceeded to surgery and underwent bilateral mastectomy. Histological examination of the left breast confirmed a 20 mm focus of grade 2 multifocal, lobular carcinoma, which was oestrogen receptor positive and Her2 receptor negative. Four out of thirty two nodes contained malignant foci. Histological examination of the right breast showed no evidence of residual disease.

Postoperatively she received radiotherapy to the left chest wall and supraclavicular fossa giving 40Gy in 15 fractions over 3 weeks. She was commenced on tamoxifen. At her most recent attendance, 3 years after her original presentation, she was well with no signs of disease recurrence and her visual acuity has improved an is now back to baseline.

## Discussion

### Central serous retinopathy

Central serous retinopathy is an idiopathic condition predominantly affecting the macula region of the retina. It is characterised by a circumscribed area of sub retinal fluid.

The pathogenesis is uncertain and postulated mechanisms include dysfunction of the retinal pigment epithelium or choroidal vascular abnormalities. [Hirami et al. 2007] Sub retinal plaques thought to be due to deposition of fibrin have been identified, suggesting increased permeability of the capillaries. [Ie et al. 1993] Indocyanine-green angiography has confirmed dye leakage from sub retinal choroidal vessels, which is thought to be due to hyperpermeability. [Piccolino and Borgia, 1994; Piccolino et al. 1995; Kitaya et al. 2003] It has also identified delayed arterial filling followed by vessel congestion, which is believed to be responsible for the development of hyperpermeable choroid. [Prunte and Flamer, 1996; Prunte, 1995]

The aetiology of this condition is unknown, but there are numerous reported associations. It more commonly affects male patients and can occur at any age. [Spaide et al. 1996] Certain personality traits, particularly the so-called “type A” personality have been suggested as a risk factor. [Yannuzzi, 1987] As in the case presented here, steroids are one of the commonly reported associations. It is reported in association both with endogenous Cushings [Bouzas, 1993] and with exogenous steroid administration. It may occur after systemic, inhaled or intraepidural steroids. [Haimovici et al. 1997; Iida et al. 2001] The effect is not thought to be dose dependent and may occur long after commencement of steroids. [Abu el Asrar, 1997; Koyama et al. 2004] It has been reported with steroid use during the treatment of haematological malignancy, for example in the treatment of Non Hodgkin’s lymphoma. [Bandello et al. 1995] There are no previous reports in association with the treatment of solid tumours. Other reported associations include, pregnancy [Sunness et al. 1993], sympathomimetic medication [Michael et al. 2003], H pylori infection [Cotticelli et al. 2006] and with sidenafil. [Allibhai et al. 2004]

This condition is usually asymptomatic unless the macula is affected in which case patients present with blurred vision, distortion or loss of visual acuity although this is usually mild. Diagnosis is based on the ophthalmoscopic findings of sub retinal fluid, and confirmed with fluorescein angiography. A characteristic “smoke stack” appearance may be seen as fluorescein leaks into the subretinal space over the course of the angiogram. Treatment is usually expectant together with the discontinuation of exacerbating factors, including steroids, if possible. In those with persistent problems photodynamic therapy may be helpful. [Cardillo et al. 2003; Taban et al. 2004; Mennel et al. 2007] As in our patient, the natural history is one of spontaneous resolution, with near full recovery of visual acuity. However the condition may recur in up to fifty percent. [Gilbert et al. 1984] In some cases it may develop into a chronic condition resulting in irreversible visual impairment. [Loo et al. 2006]

### The role of steroid premedication during docetaxol administration

Docetaxol is a semi synthetic taxane. Paclitaxol, its parent compound, was derived from Yew tree bark. Initial phase II studies using Taxanes were conducted without premedication. [Chevallier et al. 1995; Dieras et al. 1994] These studies demonstrated high overall response rates but also showed high levels of fluid retention, which was severe enough to require discontinuation of treatment in up to 50 percent. Studies comparing premedication against various regimens have also shown high levels of hypersensitivity reactions and rash in those treated without premedication. [Eisenhaur et al. 1994] A 5-day steroid regimen was shown to reduce the incidence of both hypersensitivity and fluid retention. Comparison of a 5 and 3-day regime showed that both were effective in reducing the incidence and severity of fluid retention when compared to no premedication. They also postponed the time to fluid retention and allowed a higher total dose of Taxane to be administered. This effect was more marked in the group receiving a 5-day regimen of premedication. However the 5-day group also had higher overall incidences of infection, stomatitis and diarrhoea. The study concluded that the 3-day regime had an overall better risk benefit ratio than a 5-day regime. [Riva and Lebeck, 1996] A further study examined the role of a 24-hour premedication regime in combination with antihistamine. This concluded that while it did reduce the incidence of toxicities from the taxane it was less effective in doing so than the 3-day regimen. [Fulmoleau et al. 1996] The current recommendation is therefore that all patients should be premedicated with a 3-day regime of steroids, which should be commenced 24 hours prior to chemotherapy. In the case presented here the patient was able to complete 3 further cycles on a reduced steroid regime to just 24 hours without further toxicity.

## Conclusion

A case is presented, of a woman who developed central serous retinopathy, an uncommon complication of steroid treatment which has not been previously reported in association with the treatment of solid tumours. It illustrates that it should not be assumed that high risk cancer patients who develop serious symptoms have developed metastatic disease. Clinicians should be alert to both the possibility of non malignant diagnoses in oncological patients and to the possibility of treatment related complications. With the increasing use of Taxanes in the management of solid tumours this particular treatment related complication may be seen more commonly. When such complications develop it is often necessary to make difficult decisions regarding further management on the basis of potential risk benefit ratios. In this case it was clearly vital to continue with docetaxol chemotherapy, but with a reduced dose of steroid premedication, in order to optimise her chance of visual recovery. However this was coupled with the risk that she might experience additional toxicities as a consequence of the reduction in steroid premedication, which was a challenging scenario in an understandably very anxious patient.

## Figures and Tables

**Figure 1a:**
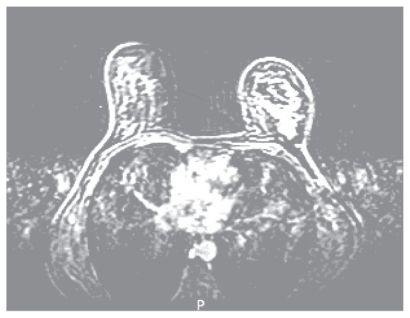
Baseline dynamic contrast enhanced breast MRI demonstrating extensive malignant type enhancement in the left upper outer breast.

**Figure 1b:**
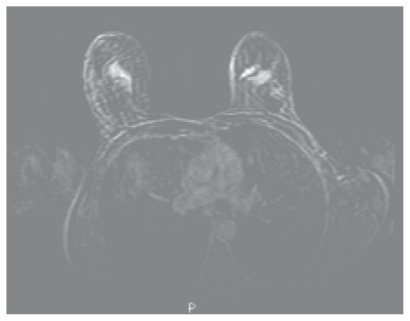
Dynamic contrast enhanced Breast MRI following 4 cycles FEC demonstrates continuing malignant enhancement in the left upper outer breast but a new enhancing lesion in the right upper outer breast, these two areas have similar malignant type contrast enhancement characteristics.

**Figure 1c:**
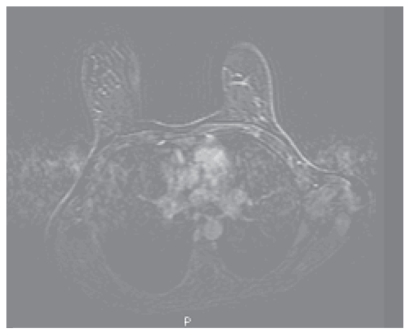
Dynamic contrast enhanced Breast MRI following 6 cycles Docetaxol shows no residual abnormal enhancement and complete resolution of the previous highly suspicious, typically malignant MR findings.

**Figure 2a:**
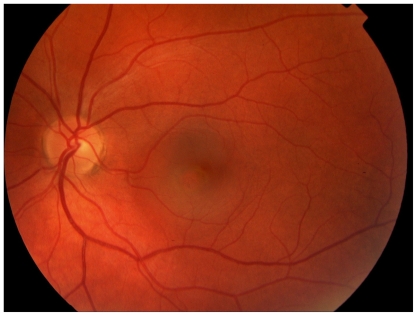
Left fundal image showing central serous retinopathy-with central pigment epithelial changes with overlying sub retinal fluid.

**Figure 2b:**
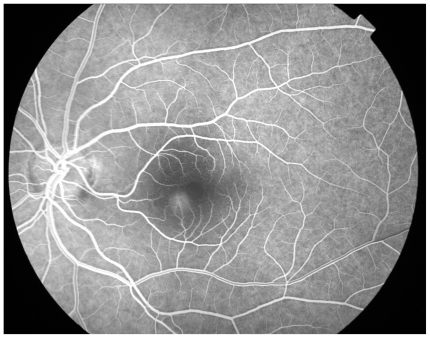
Early fluorescein angiogram showing fluorescein leak.

**Figure 2c:**
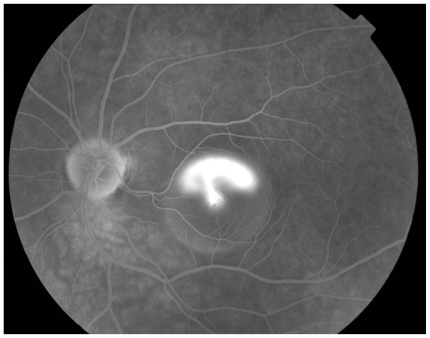
Late fluorescein angiogram showing classic “smoke stack” leakage of fluorescein and accumulation in overlying sub retinal fluid.
